# Supplementary material for: Cardiovascular disease risk among Australian unpaid carers – A survival analysis using 15 waves of the HILDA survey
Source: PLoS One. 2025 Jun 9;20(6):e0323245. doi: 10.1371/journal.pone.0323245 (PMC12148131; doi:10.1371/journal.pone.0323245)
Supplement: S1 Table — (DOCX) [file pone.0323245.s001.docx]

| **Table S1.** Baseline (wave 3) characteristics of participants with missing data and no missing data | | |
| --- | --- | --- |
| Variable | Missing data n=1,142  (%) | No missing data n=11,123  (%) |
| Gender   - Female - Male | 41.9  58.1 | 53.3  46.7 |
| Age group   - 18 to 24 - 25 to 34 - 35 to 44 - 45 to 54 - 55 to 64 - 65 to 74 - Over 75 | 15.8  18.7  17.3  18.9  10.9  6.7  11.8 | 12.8  18.7  23.1  18.9  13.2  8.4  4.9 |
| Country of birth   - Australia - Other English speaking - Non-English speaking - Australian of Indigenous origin - unknown | 35.8  4.1  13.0  2.1  44.9 | 75.1  10.5  12.6  1.8  - |
| Education   - School not completed - Finished high school - Diploma/certificate - Bachelor’s degree or higher - unknown | 25.3  8.9  13.3  7.2  45.3 | 35.1  16.0  28.3  20.6  - |
| Household structure   - Couple with no children - Couple with children - Lone parent with children - Lone person - Other | 22.2  46.1  9.0  10.1  12.6 | 29.2  41.9  8.8  15.6  4.6 |
| Long-term health condition   - Yes - No - unknown | 17.8  25.0  57.3 | 25.7  74.3  - |
| Labour force status   - Employed - Unemployed - Not in the labour force - unknown | 23.9  1.8  17.1  57.3 | 66.2  3.1  30.7  - |
| Location   - Major city - Regional - Remote - unknown | 67.7  29.7  2.5  0.1 | 62.3  35.4  2.2  - |
| Disposable income   - Quintile 1 (lowest) - Quintile 2 - Quintile 3 - Quintile 4 - Quintile 5 (highest) | 17.2  15.8  15.6  19.7  31.7 | 14.1  16.0  18.2  22.6  29.1 |
