# Supplementary material for: Cardiovascular disease risk among Australian unpaid carers – A survival analysis using 15 waves of the HILDA survey
Source: PLoS One. 2025 Jun 9;20(6):e0323245. doi: 10.1371/journal.pone.0323245 (PMC12148131; doi:10.1371/journal.pone.0323245)
Supplement: Attachment — Submitted filename: Response to Reviewers_PLOS ONE.docx [file pone.0323245.s003.docx]

**Manuscript reference number: PONE-D-23-41861**

**Cardiovascular Disease Risk Among Australian Informal Carers – A Survival Analysis using 15 waves of the HILDA Survey**

*We thank the reviewer and editors for their time and considered comments and believe that in addressing them, we have substantially improved our manuscript. Our specific responses to each of the comments are detailed below.*

**Reviewer #1:**

The paper is of reasonable quality. However, there are major changes to how the analysis is conducted and written up that have to change before it is ready for publication. I am recommending these points are comprehensively addressed before proceeding to publication.

**Comment R1.1**

Outcome measure - include the exact wording of the question. It's hard to understand the outcome in this paper without you detailing what the question is. Furthermore, are you trying to determine something equivalent to incident CVD? Is that why people with CVD at baseline were excluded.

**Response R1.1**

*Thank you for noting the ambiguity here. We have clarified that the outcome measure was incident CVD throughout the paper.*

**Comment R1.2**

Population - how many people in the younger age groups reported they had heart disease? If it is very few, I think these people should be excluded from the analysis and you should focus on the age bands where heart disease starts to emerge. I know you discuss this later in the study, but it's an important consideration in the calculation of your outcome. Including younger people increases the size of the denominator, which lowers the risk of CVD, which could impact the estimation of relative inequalities.

**Response R1.2**

Thank you for this comment. This was something that the authors considered deeply. While we recognise that CVD is something that impacts older populations, and is rare in younger groups, we also note that we were assessing outcomes across a 15-year period. At final follow up, the youngest group in our sample were in their mid-thirties. While CVD is uncommon in this age group, recent Australian Institute of Health and Welfare (AIHW) statistics evidence the fact that CVD risk starts to rise from about the age of 35, especially among men (AIHW, 2024). Therefore, we have kept our main sample as it was. However, in light of your comment, we have carried out a sensitivity analyses in which we restricted the dataset to those aged 35 years and over at baseline and found that these sensitivity results (as presented below), support the main findings. We will leave it up to the editor’s discretion as to whether these additional sensitivity results are presented as supplementary files for the paper.

*Australian Institute of Health and Welfare (AIHW). 2024. Heart, stroke and vascular disease: Australian facts, All heart, stroke and vascular disease. Canberra. Australia [Available from:* [*https://www.aihw.gov.au/reports/heart-stroke-vascular-diseases/hsvd-facts/contents/all-heart-stroke-and-vascular-disease*](https://www.aihw.gov.au/reports/heart-stroke-vascular-diseases/hsvd-facts/contents/all-heart-stroke-and-vascular-disease)*.*

| **Sensitivity analysis for those aged 35 years and over at baseline.**  Age-adjusted* estimates of incident CVD hazard ratios by caring intensity and stratified by gender | | | | | | |
| --- | --- | --- | --- | --- | --- | --- |
|  | Female | | | Male | | |
| Caring intensity | HR | 95% CI | p-value | HR | 95% CI | p-value |
| -    No caring | ref |  |  | ref |  |  |
| -    <14 hours/week | 0.75 | (0.47 - 1.19) | 0.220 | 0.89 | (0.59 - 1.36) | 0.599 |
| -    ≥14 hours/week | 1.45 | (0.94 - 2.24) | 0.094 | 1.10 | (0.65 - 1.86) | 0.722 |
| *Model adjusted for wave of observation, and age. | | | | | | |

| **Sensitivity analysis for those aged 35 years and over at baseline.**  Covariate Adjusted* estimates of incident CVD hazard ratios by caring intensity and stratified by gender | | | | | | |
| --- | --- | --- | --- | --- | --- | --- |
|  | Female | | | Male | | |
| Caring intensity | HR | 95% CI | p-value | HR | 95% CI | p-value |
| -    No caring | ref |  |  | ref |  |  |
| -    <14 hours/week | 0.78 | (0.49 - 1.25) | 0.301 | 0.89 | (0.58 - 1.36) | 0.585 |
| -    ≥14 hours/week | 1.17 | (0.76 - 1.83) | 0.475 | 0.92 | (0.54 - 1.58) | 0.770 |
| *Model adjusted for wave of observation, age, Indigenous ethnicity, country of birth, education, household structure, long-term health condition, labour force status, location, and income. | | | | | | |

**Comment R1.3**

Relative and absolute inequalities need to be presented. this could help explain why women tend to have higher relative inequalities (in the unadjusted model at least) as the baseline level of CVD among female non-carers is lower.

**Response R1.3**

Thank you for this comment. However, we note that we have carried out a survival analysis, which calculates hazard ratios (a relative measure) using time-varying measures. It is not common practice to produce measures of absolute inequalities in analysis of this kind, as survival data are time-dependent, and the risk of a CVD event can change over time. Absolute rates are unable to capture this variability and may lead to misestimation of likelihood of events at different time points.

**Comment R1.4**

Confounders - I don't understand why you haven't included a table for the confounders split by the exposure. I have no way of knowing what was happening in your adjustment.

**Response R1.4**

As we present gender stratified analysis, we have split Table 1 according to gender. To further split by exposure status would make the table infeasibly large. We are happy to take the editor’s advice on this.

**Comment R1.5**

Time varying confounders - I think this is tricky, it just feels like the variables have been dumped into the model without thinking through the causal structure. Is income a confounder? It can lead to someone in the household acquiring disability or care needs. Caring can then lead to pressure on ability to work, potentially lowering income. In this case it switches from being a confounder at time point 1 to being a mediator at time point 2. The DAG would be something like: Income -> Caring -> Income -> CVD. I think this could materially affect your results given adjustment seems to knock out a lot of the effect for women. If caring -> income, you have just conditioned on a mediator, and you are masking the impact of caring -> CVD.

**Response R1.5**

We have carefully considered the causal structure of this analysis, and the confounders included. We present the DAG that guided our conceptualisation of covariates that could be considered plausible common causes of both caregiving and CVD (based on the existing literature) below for your reference. We agree that consideration of income is not straight forward. We concur that income could be considered a mediator of the relationship between caregiving and CVD, however we also argue that it could be a determinant of caregiving, given that: a) those on low income have less means to outsource caregiving responsibilities; b) if there are alternative caregivers, persons on low income are more likely to become caregivers (selected into caregiving), because it will be deemed preferable that the higher income earner remain in the workforce. We also note that income could also plausibly be an effect modifier of the relationship between caregiving and CVD if those who are caregiving are able to “purchase” better CVD health – e.g. purchase healthier food to mitigate the effects of caregiving. Our main models include income as a confounder; however, we note that sensitivity analyses in which we excluded income from models made negligible difference and substantiated the main findings.

**Comment R1.6**

Coding of income - maybe less important, and I don't have a strong view, but thought needs to be given as to whether household income is the best measure.

**Response R1.6**

The measure of income that we use is a well-used and widely recognised measure. We have included the following justification in the manuscript (page 6, para 1):

*“Household disposable income, calculated by summing the income components for the previous financial year for all adults in the household, was equivalised using the modified OECD scale (34). For each wave of data, nominal household income values were converted to quintiles of the Australian population distribution using percentile statistics for the corresponding financial year from the Australian Bureau of Statistics biennial Survey of Income and Housing. The lowest quintile of the population distribution of disposable household income was used as the referent group (35).”*

**Comment R1.7**

Results, statistical interpretation - this is currently wrong. You haven't found there is "no difference", you just don't have sufficient evidence to reject the null hypothesis.

**Response R1.7**

Thank you for noting this. We have modified expression in the abstract:

*“These findings provide no evidence to reject the null hypothesis that providing informal care to others increases the risk of developing CVD in the Australian population.”*

And in the discussion:

*“The results of this study indicate that, in this sample of Australian adults, there was no evidence that those providing informal care to an elderly or disabled relative were at increased risk of incident CVD.”*

We have also removed statements claiming definitive findings:

*e.g. “This indicates that Australian adults who provide informal care appear to be no more or less likely to have CVD than non-carers.”*

**Comment R1.8**

Table 1 - I don't understand why there are so few observations in the 18-24 group. Is this because they didn't answer the CVD question - hence were excluded from the study.

**Response R1.8**

Thank you for bringing this to our attention. You are correct that there are slightly fewer observations in the 18–24-year age group given this age group is a small proportion of the HILDA sample and there was some missing data on CVD and caregiving in this age group. We also note that we have rerun analyses and amended Table 1 to reflect descriptive statistics at baseline (wave 3).

**Comment R1.9**

Limitations section - I think the limitations section needs to be much clearer. How does a lack of statistical power lead to underestimation of risk? It leads to lower precision. If the smaller sample size is a result of missing data that affects some groups more than others, or attrition related to the outcome or exposure, then that could lead to bias. But a small sample on its own does not necessarily lead to bias, just imprecise estimates. I think each of the separate forms of bias discussed need to be set out clearly, with an assessment of which direction they could bias the results. This could even be done using bullet points or one bias consideration per paragraph. Currently it feels a little like word soup.

**Response R1.9**

We have rewritten the limitations section to address this comment:

*“This study also had some limitations. First, generalisability of these findings to the broader Australian population may be limited since the HILDA survey under-represents people of lower socioeconomic positions, as well as migrants/new Australians (38). As all variables were self-reported, there is some risk of self-reporting bias. Given that some carers may not identify as carers, the self-reported nature of the collected variables could lead to bias due to exposure misclassification, potentially underestimating associations between caregiving and CVD. Furthermore, the measure of caregiving available in this data does not capture variations in the intensity and duration, nor type of caregiving. Notably the caregiving measure did not include those who provided caregiving for someone who is neither elderly nor has a disability, potentially underestimating true associations. Non-familial caregiver/care recipient relationships are also likely excluded by the definition of the exposure variable that was used, leading to misclassification of caregiving and underestimation of associations. Misclassification of the outcome, which was measured as self-reported CVD status, may also have occurred whereby participants may have not reported having CVD despite actually having CVD - either because they were unaware or because they did not want to disclose this information - potentially resulting in bias toward the null.*

*There is also potential for bias due to missing data, as although the final analytic sample only contained participants who had complete data for exposure, outcome, and covariates, there was a significant proportion of eligible participants with missing data for CVD status who were therefore excluded from the analysis. The demographic differences at baseline between those who were removed from the study population due to missing CVD status data and those included in the analytic sample are included as supplementary material. It is notable that among these participants from the sample due to missing data, there were more informal carers, over-65s, Indigenous Australians, people with a long-term health condition, and people in the two-lowest population income quintiles than the set of participants which comprised the sample used for analysis. Given that these factors might be associated with increased CVD risk, it is possible that this analysis may have underestimated the risk of CVD compared with what would be observed if complete data for baseline CVD status was available for all eligible participants.*

*Furthermore, this study may have been limited by a lack of statistical power due to the small number of incident CVD events observed during the follow up period. This lack of power may have been exacerbated in our gender stratified analysis, leading to imprecision in our risk estimates. Lastly, the extent to which these results may be generalisable to populations of carers in other countries is unclear. Acknowledging the limitations of this study in addressing the research question, it is clear that further research to interrogate the relationship between caregiving and CVD, with more inclusive measures of care provision and accounting for types of caregiving required (i.e., what activities require assistance, is the caregiving long-term or short-term?) and also the nature of the caregiver/care-recipient relationship (familial or otherwise).”*

**Comment R1.10**

Strengths - I am worried that what you think is a strength - how you've chucked time varying measures into the model, is in fact a weakness and is risking biasing your results. Please think this through carefully.

**Response R1.10**

We have modified this to now clarify the strength of these models in terms of well-considered covariates included as confounders:

*“Firstly, the survival analysis methods analysis to assess the effect of changes in care over time on CVD were carefully considered, with time-varying covariates selected according to a Directed Acyclic Graph as being plausible common causes of caregiving and CVD.”*

**Comment R1.11**

Have you checked how your incidence / rates of CVD compare to what is observed in other datasets? This will help you understand what you have captured in your data. Potentially use the Census to check - I think there are chronic health condition questions in the census now, the same question might have even been used!

**Response R1.11**

Thank you for your comment. To clarify, our study does not aim to estimate the incidence of cardiovascular disease (CVD). Instead, we focus on comparing the hazard ratios of experiencing a CVD event between carers and non-carers over time. This approach allows us to evaluate the relative risk of a CVD event occurring in carers compared to non-carers, rather than calculating the overall number of new CVD cases (incidence) in the population. By using a time-to-event analysis, we are able to assess the long-term hazards associated with caregiving in relation to CVD events. However, we have included further information about the prevalence and incidence of CVD in Australia in the introduction (see page 3, para 1) and below:

*It is noteworthy that the distributions of both informal caregiving and CVD in the Australian population are gendered. As previously mentioned, the informal care contribution of women outnumbers that contributed by men (7). In contrast, CVD is higher amongst men compared to women (29). In Australia, 1.2 million people aged 18 and over (6.2% of the adult population) were living with one or more conditions related to heart, stroke or vascular disease in 2017–18 (29). Based on self-reported data, the prevalence of heart, stroke and vascular disease among adults was higher among men (641,000, an age-standardised rate of 6.5%) than women (509,000, an age-standardised rate of 4.8%) (29). However, there are key distinctions in the development of CVD by gender in Australia. Whilst CVD is more prevalent in males (compared to females) over the age 45, the prevalence of CVD among females is reportedly twice that of males in the under-45 population (29). Gender differences in the severity of CVD can also be observed. For example, women are more likely than men to be hospitalised or die due to hypertensive disease, with atrial fibrillation and stroke both also causing greater mortality amongst Australian women than men (29).*
