# Supplementary material for: Cardiovascular disease risk among Australian unpaid carers – A survival analysis using 15 waves of the HILDA survey
Source: PLoS One. 2025 Jun 9;20(6):e0323245. doi: 10.1371/journal.pone.0323245 (PMC12148131; doi:10.1371/journal.pone.0323245)
Supplement: Attachment — Submitted filename: Response to Reviewers2_PLOS ONE.docx [file pone.0323245.s004.docx]

**Manuscript reference number: PONE-D-23-41861**

**Cardiovascular Disease Risk Among Australian Unpaid Carers – A Survival Analysis using 15 waves of the HILDA Survey**

*We thank the reviewers and editors for their ongoing time and expertise in reviewing our manuscript. Our specific responses to each of the remaining comments from Reviewer 3 are detailed below.*

**Reviewer #2:** The manuscript focuses on important questions and the risk of developing CVD among informal caregivers in the Australian context is under studied. The paper is well written, and I believe the authors have comprehensively addressed the detailed comments and requests from the prior review. It is notable that the HILDA dataset used in the current analysis contained a relatively large amount of missing data on CVD status among participants who were older, informal caregivers, indigenous Australians from lower income brackets and the limitation is rightly described. Further studies are therefore required to validate these findings.

**Response:** *We thank Reviewer 2 for their time and expertise in reviewing our manuscript and their positive comments.*

……………………………………………………………………………………………………………

**Reviewer #3:** This review evaluates the manuscript titled “Cardiovascular Disease Risk Among Australian Informal Carers – A Survival Analysis using 15 waves of the HILDA Survey”. The study explores the relationship between informal caregiving and the risk of cardiovascular disease (CVD), given the potential stress associated with caregiving. However, the authors did not find any evidence suggesting an increased risk of CVD due to informal care.

**Comment R3.1**

The research topic is engaging and relevant; however, the study’s findings do not support the hypothesized link between stress from caregiving and CVD risk. My primary concern lies in the study design, as it appears the analysis was tailored to the research question, rather than the research question driving the analysis.

**Response R3.1**

*We thank reviewer 3 for their time in reviewing our manuscript and concur that the study’s predominantly null findings do not support the hypothesised link between the stress associated with caregiving and CVD. Nonetheless, we believe our findings to be robust. We do not consider that null findings should preclude publication, and indeed we argue that to do so would contribute to publication bias.*

*That said, we note that revised analyses conducted in accordance with the reviewer’s suggestions support a more nuanced interpretation of the association between unpaid caregiving intensity and incident CVD among females Our results have been updated accordingly.*

*As a further point, we reject the reviewer’s implication that we did not conduct this research according to the highest research principles. The study in question directly succeeded and was based on a comprehensive systematic review of the literature that we performed (and published) in 2023.* The findings of the review subsequently led to a very specific research question regarding the Australian caregiving population which then drove our choice of analysis and methodology.*

*** *Lambrias, A., Ervin, J., Taouk, Y., & King, T. (2023). A systematic review comparing cardiovascular disease among informal carers and non-carers. Int J Cardiol Cardiovasc Risk Prev, 16, 200174. doi:10.1016/j.ijcrp.2023.200174*

**Comment R3.2**
The outcome variable, CVD, reflects a heterogeneous population. For instance, the inclusion of participants as young as 18 years old introduces variability, particularly since the youngest participants in the later waves are only 33 years old, an age group with a relatively low likelihood of CVD incidence. Additionally, unpaid caregiving intensity and associated stress levels vary widely based on individual circumstances and the nature of care, further contributing to data heterogeneity. This high heterogeneity likely increases variance, which can make it difficult to reject the null hypothesis.

**Response R3.2**

*Thank you for this comment. This was something that the authors considered deeply. Further to our initial response (pasted below for reference), we acknowledge the significant (and expected) heterogeneity of our sample population. As with all research, there are limitations to this analyses, and we acknowledge this in the limitations.*

*Whilst we fully appreciate that caregiving intensity and associated stress levels vary widely based on individual circumstances and the nature of care, both unpaid caregiving and the outcome (CVD) are relatively uncommon (~10% of our sample were caregivers and there were only 686 cases of heart disease in our 11,123 participants). Therefore, in order to maintain sufficient statistical power to examine the broader question/association in question; we sought to include as many exposed individuals as possible over the 15 year follow up period, noting that we did conduct analysis of two different levels of caregiving intensity (greater or lesser than 14 hours/week caring).*

| **Table 1.** Descriptive statistics of analytic sample | | | |
| --- | --- | --- | --- |
|  | Female | Male | Total |
| Number (%) of participants | 5,927 (53.3%) | 5,196 (46.7%) | 11,123 |
| Mean (SD) follow-up time (years) | 11.94 (4.75) | 11.69 (4.84) | 11.82 (4.79) |
| Cases of heart disease reported | 326 | 360 | 686 |
| Caregiving (%)   - None - <14 hours/week - ≥14 hours/week | 87.6  8.8  3.6 | 91.4  6.3  2.3 | 89.4  7.7  3.0 |
| Baseline Characteristics at wave 3 | | | |
| Age in years Mean (SD) | 44.51 (16.69) | 43.80 (16.11) | 44.48 (16.42) |

*Regarding your comment about the younger adults in our sample, we note that the number of 18–24-year-olds is only 13% of the sample, with the mean age of our sample at baseline being 44 years. Furthermore, the sensitivity analysis that we conducted on a sample restricted to those 35 years and over supported out main findings (see below).*

*(Initial response to comment in Review #1) While we recognise that CVD is something that impacts older populations, and is rare in younger groups, we also note that we were assessing outcomes across a 15-year period. At final follow up, the youngest group in our sample were in their mid-thirties. While CVD is uncommon in this age group, recent Australian Institute of Health and Welfare (AIHW) statistics evidence the fact that CVD risk starts to rise from about the age of 35, especially among men (AIHW, 2024). Therefore, we have retained our main sample. However, in light of this comment, we have carried out a sensitivity analyses in which we restricted the dataset to those aged 35 years and over at baseline and found that these sensitivity results (as presented below), support the main findings.*

*Australian Institute of Health and Welfare (AIHW). 2024. Heart, stroke and vascular disease: Australian facts, All heart, stroke and vascular disease. Canberra. Australia [Available from:* [*https://www.aihw.gov.au/reports/heart-stroke-vascular-diseases/hsvd-facts/contents/all-heart-stroke-and-vascular-disease*](https://www.aihw.gov.au/reports/heart-stroke-vascular-diseases/hsvd-facts/contents/all-heart-stroke-and-vascular-disease)*.*

| **Sensitivity analysis for those aged 35 years and over at baseline.**  Age-adjusted* estimates of incident CVD hazard ratios by caring intensity and stratified by gender | | | | | | |
| --- | --- | --- | --- | --- | --- | --- |
|  | Female | | | Male | | |
| Caring intensity | HR | 95% CI | p-value | HR | 95% CI | p-value |
| -    No caring | ref |  |  | ref |  |  |
| -    <14 hours/week | 0.78 | (0.49 - 1.24) | 0.297 | 0.87 | (0.57 - 1.33) | 0.518 |
| -    ≥14 hours/week | 1.47 | (0.95 - 2.27) | 0.084 | 1.00 | (0.58 - 1.72) | 0.995 |
| *Model adjusted for wave of observation, and age. | | | | | | |

| **Sensitivity analysis for those aged 35 years and over at baseline.**  Covariate Adjusted estimates of incident CVD hazard ratios by caring intensity and stratified by gender | | | | | | |
| --- | --- | --- | --- | --- | --- | --- |
|  | Female* | | | Male** | | |
| Caring intensity | HR | 95% CI | p-value | HR | 95% CI | p-value |
| -    No caring | Ref |  |  | ref |  |  |
| -    <14 hours/week | 0.81 | (0.51 - 1.29) | 0.374 | 0.86 | (0.56 - 1.32) | 0.504 |
| -    ≥14 hours/week | 1.18 | (0.76 - 1.84) | 0.456 | 0.83 | (0.48 - 1.45) | 0.517 |
| *Model adjusted for wave of observation, age, Indigenous ethnicity, country of birth, education, household structure, labour force status, location, and income, and stratified by long term health condition.  **Model adjusted for wave of observation, age, Indigenous ethnicity, country of birth, education, household structure, long-term health condition, labour force status, location, and income. | | | | | | |

**Comment R3.3**
Although the authors conducted sensitivity analyses on limited subpopulations, they employed the same methodology without adequately addressing the heterogeneity. Before conducting specific analyses such as survival analysis, I would recommend basic statistical investigations to better understand the data. For example:
• Perform bivariate analyses to examine relationships between age groups, CVD incidence, informal caregiving intensity, and stress levels.
• Investigate high-risk groups, such as middle-aged single mothers with caregiving responsibilities and high K10 scores, to determine their CVD rates.
• Use crosstab analyses to assess CVD rates among subgroups with informal caregiving responsibilities and varying stress levels.
• Validate these findings using general logistic regression before progressing to survival analysis, ensuring the analysis is warranted and appropriately contextualized.
By addressing these foundational issues, the study could more robustly address the research question and manage the heterogeneity in the data.

**Response 3.3**

*We thank the reviewer for their thoughtful feedback and suggestions to improve our analysis. Below, we address the specific recommendations and provide a rationale for not incorporating them into the current study.*

*Bivariate Analyses of Key Relationships: While we understand the importance of bivariate analyses in exploring relationships between variables, the purpose of our study was to assess the risk of caring over time on CVD. Bivariate analyses, though useful for descriptive purposes, do not account for confounding factors and could provide misleading results if interpreted without considering the broader context. Our multivariate survival analyses were designed to adjust for potential confounders and interactions, providing a more robust understanding of these relationships. As such, we believe that performing bivariate analyses would not add value to the conclusions already supported by our primary analyses, and would unnecessarily blow out the journal word count.*

*Investigating High-Risk Groups: We agree that identifying high-risk groups is important for targeted interventions; however, the scope of our study was to examine broader population-level patterns rather than focus on specific subgroups. Additionally, we lacked sufficient statistical power to conduct subgroup analyses (e.g. stratifying by variables such as age would lead to small sample sizes in some strata, drastically reducing statistical power). Instead, we addressed heterogeneity in the dataset by including interaction terms and stratified analyses where appropriate. These approaches allowed us to assess how the effects of caregiving intensity vary across demographic subgroups without overextending the scope of the study.*

*Crosstab Analyses of CVD Rates: Crosstab analyses are often helpful for descriptive studies which we describe in detail in “Table 1: Descriptive statistics of analytic sample”. However, our study focused on modelling associations rather than purely descriptive relationships. Cross-tabulations, while intuitive, are limited in their ability to account for confounding and do not align with our analytical objectives. Our survival analyses were specifically designed to incorporate covariate adjustments, providing a more comprehensive understanding of the impact of caregiving on CVD while adjusting for other factors influencing CVD incidence. Including crosstab analyses would therefore be redundant and unlikely to contribute novel insights.*

*Validation with Logistic Regression Before Survival Analysis: Logistic regression is indeed a useful preliminary tool for evaluating associations; however, our primary research question necessitated survival analysis to account for time-to-event data and censoring since we were interested in the development of CVD over time. The use of survival analysis inherently incorporates the strengths of logistic regression while providing additional temporal insights. To ensure the robustness of our findings, we conducted specific diagnostic checks, e.g., proportional hazards testing, and sensitivity analyses. These confirm that our survival models were appropriate and adequately addressed the research question without the need for preliminary logistic regression.*

*We appreciate the reviewer’s suggestions and agree that these analyses could be useful in certain contexts. However, given the study’s objectives, our existing methods effectively and efficiently address the research question. Simultaneously, our methods account for heterogeneity and ensure robust, generalisable findings. Incorporating the suggested analyses would be outside the scope of this study and potentially redundant given the comprehensive nature of our current approach.*

**Comment 3.4**. There is inconsistency in how the waves and years of data are described. My understanding is that the authors used waves 3, 7, 9, 13, and 17. However, the manuscript implies data spanning waves 3–17. This should be clarified and corrected.

**Response 3.4**

*Thank you for this opportunity to clarify the waves used. Data spanning waves 3-17 was used in the study. This is because caregiving exposure is collected annually. As such, data spanning waves 3-17 was used to assess caregiving status and then incident CVD was measured at every wave it was available (which was waves 3, 7, 9, 13, 17). Self-reported CVD status was first measured in wave 3 (2003) of the HILDA survey and for this reason it was selected as the baseline time at which participants must be active in the study. Likewise, follow-up continued until wave 17 (2017), the most recent wave at which CVD status was measured. In this way CVD status was collected at both ends of the time period included in this analyses. Moreover, caregiving exposure could precede the CVD outcome in the years leading up to the next CVD outcome wave. To clarify this further, we have added an additional sentence to the revised manuscript when explaining our incident CVD outcome variable (see page 6, para 1).*

**Comment 3.5.** The analysis appears incomplete. Additional analyses addressing the heterogeneity and variability in the dataset are necessary to strengthen the study. A missing data analysis is relevant.

**Response 3.5**

*We conducted additional analyses to address heterogeneity and missing data. Specifically, we tested for heterogeneity in all variables included in the survival analyses using a global test of proportional hazards. This test assessed whether Schoenfeld residuals were correlated with time, which would indicate a violation of the proportional hazards assumption and suggest heterogeneity. No evidence of heterogeneity was found for caring intensity or any other variables for either males or females, except for long-term health conditions in females. As a result, we stratified the models by long-term health conditions for females to account for this heterogeneity in the survival analyses. Consequently, we have updated the tables and text in the manuscript to reflect these changes in the analyses.*

*We further investigated the missing data by describing the characteristics of participants with missing data at baseline who were therefore excluded from the analyses in Supplementary Table S1. We also compared them with the characteristics of included participants. Given the relatively low proportion of missing data (9.4%) and the analysis presented in Table S1, which supports data missing at random, we conducted a complete case analysis. This additional information has been incorporated into the manuscript, and Table S1 has been updated accordingly.*

*Additionally, we explored missingness using the Missing Indicator Method (MIM). Since most missing data stemmed from the primary exposure of interest, caregiving intensity, we created an additional level for missing values in the caregiving variable and repeated the survival analyses. However, adjusting for missingness did not alter the results. The hazard ratio for the missing values level, compared to no caregiving, was 0.0000, with no other parameters estimable across all models. As such we have decided not to include these additional analyses in the paper.*

**Comment 3.6.** The literature review should be updated to include recent research on Australian informal care, particularly studies focusing on care for disabled or elderly individuals.

**Response 3.6**

*We have conducted an updated review of the literature and are confident that our literature review is up to date. As stated in our manuscript, there is very little research in this area – this manuscript represents a robust contribution to this body of literature.*
